# Level of patient safety culture among public healthcare professionals in Pretoria

**DOI:** 10.4102/safp.v65i1.5640

**Published:** 2023-05-19

**Authors:** Tombo Bongongo, Indiran Govender, Shango N. Olowa, Nyundu S.J. Phukuta, Doudou K. Nzaumvila

**Affiliations:** 1 Department of Family Medicine and Primary Health Care, Faculty of Medicine, Sefako Makgatho Health Sciences University, Pretoria, South Africa

**Keywords:** assessment, patient safety culture, healthcare professionals, Pretoria, South Africa

## Abstract

**Background:**

Patient safety culture (PSC) norms set within an organisation prevent harm during medical care. This study assessed the level of PSC among public healthcare professionals (HCPs) in Pretoria, South Africa.

**Methods:**

A multi-centre cross-sectional study conducted in three hospitals and 25 clinics in regions 1 and 2 of Pretoria, using a self-administered questionnaire adapted from the Hospital Survey on Patient Safety Culture. Using the Raosoft online sample size formula, from 1238 public HCPs identified, the sample size was calculated at 294; this expanded to 319 as a result of respondents’ willingness to participate in the study.

**Results:**

Of the 319 respondents with a mean age of 39.9 years, the minimum and maximum ages were 22 and 66 years, respectively. The age group of 30–39 years had the highest participation rate (17.6%). Most respondents (41.1%) came from the Odi district hospital and there were more women (78.1%) and nurses (49.2%). Positive attitudes were found for all PSC components, with staff education and training scoring highest (98.7%). Patient safety culture received a satisfactory rating from HCPs from the targeted facilities.

**Conclusion:**

This study showed that public HCPs in Pretoria’s regions 1 and 2 have a good PSC, particularly among nurses, professionals with more experience, and at primary care level.

**Contribution:**

To maintain or increase awareness of this concept among HCPs, the study advocates a PSC programme as well as ongoing education that can be supported by district and facility managers.

## Introduction

Patient safety culture (PSC) might not be fully understood without mentioning ‘patient safety’, which appears to have its roots in the push for high-quality healthcare.^1^ According to the Institute of Medicine, prevention of harming a patient is the main focus when referring to patient safety.^1^ Patient safety culture tries to stop and lessen risks, mistakes and harm that happen to patients when receiving medical care.^2^ Through the concept of patient safety, various elements have been identified, such as the delivery system of care, which has to prevent mistakes and the need to learn from past mistakes. Therefore, a culture of ensuring the safety of patients has to be built and considered by all health organisations and healthcare professionals (HCPs), as well as the beneficiaries of the healthcare.^2^ Healthcare professionals and patients alike should be aware of the need to develop a PSC in a world where incapacitating injuries and deaths directly related to medical care are seen as a significant public health concern. Patient safety culture should be understood as the perceptions, attitudes or standards established within an organisation regarding the safety of patients.^3^

A Saudi Arabian study found that PSC cannot stand alone and a number of factors need to be considered while seeking to develop the PSC in a particular healthcare organisation, which can make the PSC stronger or weaker. Strong teamwork, a supportive administration, and support from the leadership team may help to prepare and enhance a PSC among the healthcare workers. Poor communication, ineffective leadership, a culture of blame (where there is no encouragement), and other factors hamper this culture.^4^ While providing care to patients in developed countries, it was observed that one patient out of 10 suffers harm, despite the fact that almost half of these cases could be avoided if PSC was institutionalised. This was noticed in light of the fact that adverse events associated with unsafe healthcare appear to be a cause of death and disability worldwide. In addition, a large number of cases include the diagnosis, prescription, and usage of medications.

Reducing patient harm or making investments in PSC could result in significant financial savings compared with the healthcare costs associated with adverse occurrences.^2^ It has been reported that in industrialised and underdeveloped nations, respectively, seven and 10 out of every 100 inpatients suffer infections related to health treatment. Up to one out of four patients who undergo unsafe procedures experiences problems, and about a million patients experience death during or shortly after surgery.^2^ Over 50% of diagnostic mistakes have the potential to be very harmful, and patients may endure misery for the rest of their lives as a result of incorrect diagnosis. Five percent of the time, this happens in outpatient settings.^2^ Infections and the likelihood of harmful transfusion reactions are two consequences of unsafe transfusion techniques. An average incidence of nine serious responses per 100 000 distributed blood components was recorded from a group of 21 nations.^2^ An analysis of research on the outcomes of questionnaires on PSC conducted in various hospitals in Arab nations revealed that the lack of communication as a factor linked to PSC is the main barrier preventing healthcare staff from reporting accidents. In such an environment, the cornerstone of patient safety will therefore be the creation of a PSC, to which all parties involved (health managers, policymakers and other leaders) need to be committed.^5^ In the same region, more in the Middle East, Jordan was found to have a moderate level of PSC, according to a survey conducted among nurses.^6^ This notion can be boosted and improved by improving related areas or factors, such as open and honest communication, staffing, handoff and transition, non-punitive solutions to errors, and teamwork, which improve patient safety, which is crucial for a better therapeutic outcome.^6^ In an Iranian study that aimed to determine and compare the views of medical staff regarding the PSC and the influence of effective features in public and private hospitals, about 70% of respondents were women and 27% were men. Findings indicated that more than 60% and more than 50%, respectively, of affirmative responses to the safety culture questionnaire were obtained from public and private organisations. The study revealed the advantages and disadvantages of the PSC; hence, appropriate measures must be developed to strengthen the concept.^7^

In Europe, a mixed-methods study carried out among nurses in nations including Croatia, Spain, Sweden, and Hungary examined perspectives on PSC.^8^ It transpired that an underdeveloped health system with insufficient leadership creates a barrier to the formation of a PSC capable of preventing and averting serious adverse events. Although PSC has been examined around the globe, very little has been reported in the European region regarding the relationship between the concept and the clinicians’ perceptions. Given the culture or variation of the health system architecture demonstrated in different countries, study of these European countries’ PSC indicated the need for further development of a PSC.^8^

In Ethiopia, working in a primary hospital was associated with PSC, whereas being between the ages of 25 and 34 years did not demonstrate an association with PSC. As there was a low level of PSC across the board in the country, it was recommended that PSC initiatives be started across the nation.^8^ A similar study on PSC and related factors was conducted in a different region of the same country and it confirmed the previous findings that there was a low level of PSC. This time, communication, teamwork, structural learning, and conversations about feedback, particularly when it was negative, working hours per week, as well as reporting of adverse events, were meaningfully associated with the studied concept. Therefore, it was recommended that a well-integrated programme must be implemented.^9^ Contrary to the Ethiopian experience, frontline healthcare workers in Yaoundé, Cameroon, showed a high level of knowledge and interest in learning about the concept of PSC.^10^ However, key problems with the culture are unsatisfactory cooperation between management and medical staff, with a low commitment to the matter from hospital authorities.^10^ In addition, the trauma PSC in Cameroon needs to change because low percentages of positive responses observed for non-punitive solutions to mistakes made by healthcare workers pointed to the necessity for a culture where blaming certain professionals may address structural problems or deficiencies in patient care.^11^ In the neighbouring country of Nigeria, more female nurses who are between the ages of 30 and 39 years and have worked in a hospital setting for at least 5 years have shown that patient harms can be minimised through intervention, but three other factors (staffing, non-punitive response to mistakes, and the number of reports of events that are happening) that need improvement, even though they are all associated with the studied concept.^12^

In South Africa, when the Manchester PSC framework questionnaire was used in a district hospital in Bloemfontein, Free State province, the nurses seemed to lack a PSC, and the doctors had unfavourable opinions of all patient safety-related factors.^13^ Health managers are therefore required to integrate a PSC into high-quality patient care.^13^ In another qualitative study carried out in South Africa examining how the strategic and operational management of various tertiary academic hospitals perceived the PSC, it was found that there is still room for improvement, despite mentioning factors such as implementation, obstacles, management and enhancing PSC in their facilities.^14^ In order to create a model of the PSC, a multi-method approach that included a historical analysis of the concept in South Africa and a literature assessment of the PSC in sub-Saharan African obstetric surgery were studied in Cape Town in the Western Cape province of South Africa as a theoretical foundation, taking into account Schein’s model of organisational culture.^15^ As a result of resource limitations and a history of discrimination in medicine, there are currently obstacles to PSC in the country, where the history of prejudice and resource shortages continue to influence current PSC. It is therefore crucial to significantly adjust or work on safety compliance and other related factors in order to attain a PSC in the country.^15^ While targeting South African tertiary academic hospitals, a qualitative study that explored managers’ impressions of the idea found that they were the most suitable to apply and ameliorate the PSC at this level of care; it also explored various issues that emerged, including a summary of the concept, its running, execution, hitches and suggestions to improve it in an academic hospital setting.^14^ Regarding this concept of PSC, this research concluded that several areas (including basic services, infrastructure and staff attitudes) needed to be improved.^14^ It is advised that more research should be performed in order to boost this culture.^14^ In light of the given considerations, this study sought to assess the level of PSC among public HCPs in the Tshwane district of South Africa.

## Methods

**FIGURE 1:**
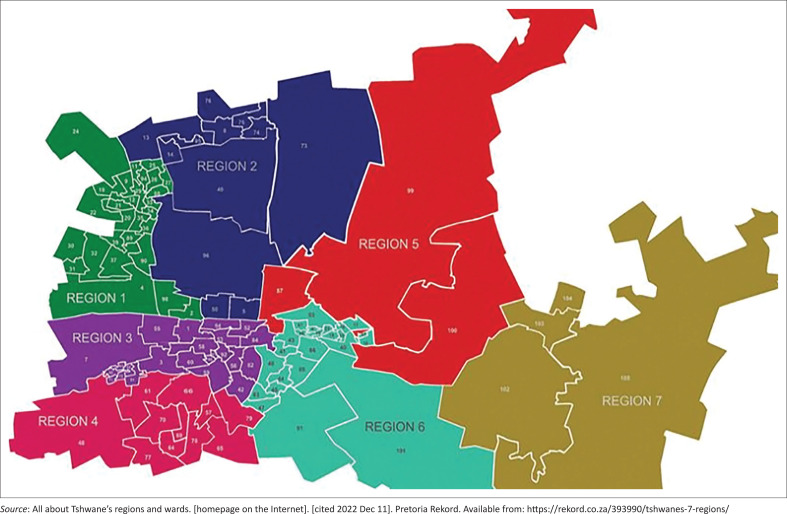
Map of all seven regions of Tshwane (Pretoria) highlighting region 1 in green and region 2 in blue.^17^

This multi-centre cross-sectional study was conducted in three hospitals (Dr George Mukhari Academic Hospital [DGMAH], Odi district hospital and Jubilee district hospital) and 25 clinics in regions 1 and 2 of Pretoria (DGMAH cluster), as shown in Figure 1^17^, using a self-administered questionnaire adapted from the Hospital Survey on Patient Safety Culture (HSOPSC), which has been used in China (2013)^16^ and Ethiopia (2021).^3^

This study was conducted in the Gauteng province of South Africa, specifically in regions 1 and 2 of the Tshwane district. Region 1 is situated in the northwest corner of the main town, named Tshwane (Pretoria). The R80-Mabopane Highway is the name of the major road that connects the central town to this region, while region 2 is bounded to the south by the Magaliesberg mountain range and to the west by PWV9 highway. The national road N1 connects it to central town.

The target demographic included all public HCPs (nurses, medical doctors, clinical associates, dietitians, occupational therapists, pharmacists, psychologists, physiotherapists, radiographers, speech therapists and social workers) employed in regions 1 and 2 of the Tshwane district, specifically in the three hospitals: DGMAH (specifically family medicine ward, family practice unit and casualty unit), Odi district hospital, Jubilee district hospital, and 25 clinics (including community health centres), and numbered 1238. The inclusion criteria included any public HCPs who were aged 18 years or older, employed in regions 1 and 2 of the Tshwane district, present during data collection, and willing to take part in the study. By using the sample size Raosoft^18^ online calculator with a margin error of 5% and a confidence interval of 95%, the estimated sample size was calculated to be 294. However, the sample size was adjusted to 319, an 8.5% increase, because of the high number of participants who wished to take part during the data collection, based on convenience sampling.

Four community health workers were selected and trained as fieldworkers by the researcher on how to explain the study’s aim and objectives, how to fill out the adapted self-administered questionnaire, and how to collect the completed questionnaires. Two fieldworkers were assigned to each region with the goal of streamlining the data collection process and reducing workload. A self-administered questionnaire was given to participants who gave their informed consent and the data collection period ran from July 2019 to February 2020.

The questionnaire used for data collection was adapted from the HSOPSC used in China and later in Ethiopia, among others, when planning and evaluating PSC. However, the tool was standardised or evaluated for the local context before being used in the Tshwane study. Socio-demographic information and an assessment of the nine components associated with PSC, such as commitment, priority, perception of the causes of incidents and identification, investigation, organisational learning, communication, personal management, staff education and teamwork were included in the questionnaire. Respondents ticked ‘Yes’ next to statements of associated components when they recognised applying or supporting them, and ‘No’ when they did not support or apply it, and ‘Not sure’ when they were uncertain. The questionnaire was piloted in the neighbouring district of Madibeng, part of the North West region of South Africa; this helped to avoid data contamination, as none of the respondents were from this province. After the pilot study, the questionnaire was modified by the authors in accordance with the aim and objectives of the study. Three medical specialists who are knowledgeable about research evaluated and corrected the questionnaire before it was used in the Tshwane study.

Descriptive analysis of the data was performed using the Statistical Package for the Social Sciences (SPSS) version 28 after the raw data had been entered into an Excel spreadsheet and imported. The descriptive analysis of the responses from the various healthcare categories establishes the mean score of responses at 6, considering the nine components or aspects that support the PSC, where 0 to 5 was rated as poor, and from 6 to 9 was rated as good.

### Ethical considerations

The Chief Executive Officers of the three hospitals (Odi, Jubilee, and Dr George Mukhari Academic Hospital), Operational Managers of clinics, and the Tshwane Research Ethics Committee (NHRD Ref number: GP_202001_048) all gave their approval for this research study. Through the Sefako Makgatho Health Sciences University Research Ethics Committee (SMUREC), Sefako Makgatho Health Sciences University provided an ethical clearance certificate (SMUREC/M/197/2019: IR). All individuals who agreed to participate signed an informed consent form, and anonymity and confidentiality were maintained during the entire research process. The principles of beneficence, nonmaleficence, autonomy and justice as fundamental to ethics were considered and upheld in this study.

## Results

Several participants did not complete the questionnaire in full as required. Because the study used a self-administered questionnaire, some data were found to be missing during the analysis, which resulted in the sum of numbers for the various socio-demographic components not being equal and displaying discrepancy.

Most participants (*n* = 56; 17.6%) were in the age group 30–39 years, with Odi district hospital having the most participants (*n* = 131; 41.1%); more women participated (*n* = 249; 78.1%), more nurses (*n* = 153; 49.2%), and more respondents with 5 to 9 years of experience (*n* = 82; 25.7%), as illustrated in Table 1.

**TABLE 1:** Socio-demographics of the healthcare professionals.

Variables	Frequency	Percentage†
**1. Age groups (*n* = 166)**		
20–29 years	29	9.1
30–39 years	**56**	**17.6**
40–49 years	46	14.4
≥ 50 years	35	11.0
Total	166	52.1
**2. Workplace (*n* = 217)**		
Clinics	99	31.0
Dr George Mukhari Academic Hospital	16	5.0
Jubilee district hospital	71	22.3
Odi district hospital	**131**	**41.1**
Total	317	99.4
**3. Gender (*n* = 316)**		
Male	67	21
Female	**249**	**78.1**
Total	316	99.1
**4. Category of HCP (*n* = 310)**		
Clinical associate	7	2.2
Dietitian	6	1.9
Medical doctor	34	10.7
Nurse	**153**	**49.2**
Occupational therapist	11	3.4
Pharmacist	7	2.2
Physiotherapist	7	2.2
Psychologist	1	0.3
Radiographer	4	1.3
Speech therapist	3	0.9
Social worker	1	0.3
Other	76	23.8
Total	310	97.2
**5. Years of experience (*n* = 317)**		
0–4 years	73	22.9
5–9 years	**82**	**25.7**
10–14 years	64	20.1
15–19 years	38	11.9
20–24 years	22	6.9
25 and more	38	11.9
Total	317	99.4

Note: Data in bold represents the highest frequencies and percentage for each variable.

HCP, healthcare professional.

† , Based on the sample size of *n* = 319.

The responses of HCPs regarding the factors associated with PSC were divided into two groups, based on the mean score of the responses, with a minimum score, and maximum score, which were 6, 0 and 9, respectively. Positive or ‘Yes’ responses were categorised as a good PSC, whereas negative attitudes were derived from the ‘No’ and ‘Not sure’ responses. All the associated factors had a high frequency and percentage of ‘Yes’ responses compared with negative responses. The ‘Yes’ responses supported a good PSC; therefore, a favourable attitude towards the concept was exhibited by the HCPs of regions 1 and 2 of the Tshwane health district, as shown in Table 2.

**TABLE 2:** Assessment of factors associated with patient safety culture in regions 1 and 2.

Factors supporting patient safety culture	Patient safety culture					
	Yes		No		Not sure	
	*n*	%	*n*	%	*n*	%
Commitment to patient safety (*n* = 311)	197	61.8	87	27.3	27	8.5
Priority given to patient safety (*n* = 316)	248	77.7	52	16.3	16	5.0
Perception of causes of patient safety incidents and their identification (*n* = 316)	257	80.6	24	7.5	35	11.0
Investigating patient safety incidents (*n* = 316)	227	71.2	45	14.1	44	13.8
Organisational learning following patient safety incidents (*n* = 312)	186	58.6	69	21.6	56	17.6
Communication about safety issues (*n* = 314)	213	66.8	56	17.6	45	14.1
Personal management and safety issues (*n* = 315)	206	64.6	75	23.5	34	10.7
Staff education and training about safety issues (*n* = 315)	193	98.7	91	28.5	31	9.7
Teamwork on patient safety issues (*n* = 311)	182	57.1	71	22.3	58	18.2

**FIGURE 2:**
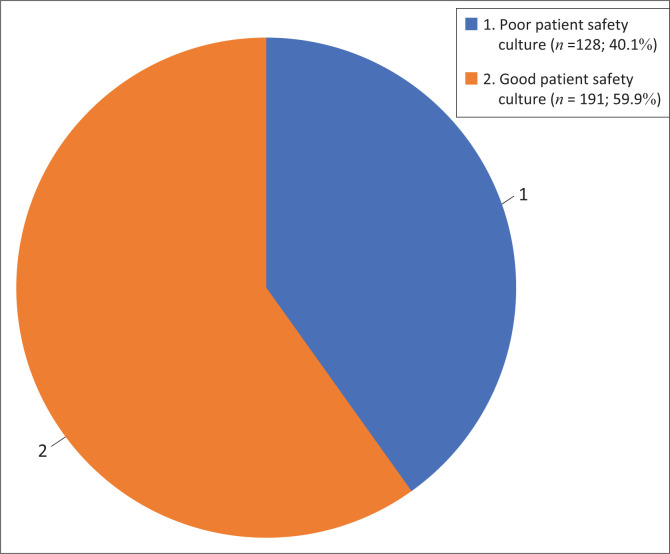
Overall patient safety culture.

While grouping HCPs’ responses from Table 2, although the number of participants with a poor PSC is quite high (40.1%), the overall outcome or more replies, if the absolute value of the two numbers is considered, were favourable for a good PSC, as presented in Figure 2.

## Discussion

While supporting a dedication to zero harm, a strong and comprehensive safety programme must be overlaid on certain existing cultural principles, such as respect, honesty, ethics, a shared sense of duty, and others. These cultural principles were demonstrated by HCPs from the two regions (1 and 2) of the Tshwane district. Targeting or providing opportunities to HCPs with less than 4 years of experience, medical doctors, other medical fraternities (with the exception of nurses), as well as tertiary institutions in order to educate them on the concept of PSC should be prioritised, because these categories of HCPs as well as tertiary institutions were rated lower regarding PSC in the current Tshwane survey.

From a demographic standpoint, the sample’s high proportion of women (nearly two-thirds of the sample) seems to be consistent with the Iranian survey, which compared public and private health facilities based on factors that influence PSC. This can possibly be explained by the sampling method used in both studies, which was convenience sampling. The same level of PSC was also observed in Iran when considering its private sector. This consistency was especially apparent in two elements that strengthen the concept: organisational learning and teamwork.^7^ Such results should be expanded to other areas of the concept in order to increase the level of PSC in the public and private sectors of the two countries. Without going into specifics, because that was outside the scope of the objectives, primary health care facilities in Tshwane and Ethiopia^3^ have demonstrated that the environment may play a key influence in developing a culture of patient safety. The aforesaid consistency regarding the PSC in primary health care has been demonstrated in Tshwane as well as in other nations such as Beirut, Lebanon, and the United States.

As a result, studies need to be initiated regarding the factors associated with good performance on PSC at this level of care while the tertiary or academic level institutions are still struggling. A contradiction has emerged at tertiary or academic institutions, where teamwork and organisational learning and growth are both positive areas, yet there is still room for strengthening areas of strength and addressing areas of weakness, as stated in a study in Riyadh.^19^ This result shows that tertiary institutions should adopt a culture of patient safety, because doing so will reduce the costs of healthcare related with unfavourable events.^2^ In Tshwane, the older generations acquire more maturity, with some disparities in the culture around patient safety. This Tshwane outcome does not align with the results of other African investigations.^3^ This inconsistency might be caused by the lack of a PSC in various age groups, so implementing a programme on the concept’s culture could raise PSC levels across age groups. The PSC score of 58% for nurses in this study, which is higher than that of medical doctors and other medical fraternities, is consistent with descriptions from other parts of the world (Vietnam),^20^ where the average scores for the PSC components such as ‘staffing’, ‘management support for patient safety’, ‘teamwork across units’, and ‘handoffs and transitions’ were considerably higher among nurses than among physicians.^20^ Such an outcome may be attributed to the awareness of this culture in the nursing fraternity.

### Strengths and limitations

This result cannot be generalised, because not all HCPs in the two regions (1 and 2) of the Tshwane district participated because of the self-administered nature of the questionnaire.

## Conclusion

This study has shown that public healthcare workers in Tshwane’s regions 1 and 2 have a good PSC, and that it is particularly positive among nurses, professionals with more experience, and at the primary care level. Thus, it is crucial to promote a PSC programme and continuing education that can be supported by the district and facility managers, in order to maintain or raise awareness of this concept among HCPs.
